# The CB1 cannabinoid receptor regulates autophagy in the tibialis anterior skeletal muscle in mice

**DOI:** 10.1186/s40659-023-00426-5

**Published:** 2023-03-25

**Authors:** Carlos Sepúlveda, Juan Manuel Rodríguez, Matías Monsalves-Álvarez, Camila Donoso-Barraza, Francisco Pino-de la Fuente, Isabelle Matías, Thierry Leste-Lasserre, Philippe Zizzari, Eugenia Morselli, Daniela Cota, Miguel Llanos, Rodrigo Troncoso

**Affiliations:** 1 Laboratorio de Investigación en Nutrición y Actividad Física (LABINAF), Instituto de Nutrición y Tecnología de los Alimentos (INTA), Universidad de Chile, Santiago, Chile; 2 Laboratorio de Ciencias del Ejercicio, Clínica MEDS, Santiago, Chile; 3 Universidad de O’Higgins, Rancagua, Chile; 4 Advanced Center for Chronic Diseases (ACCDiS), Universidad de Chile, 8380492 Santiago, Chile; 5 University of Bordeaux, INSERM, Neurocentre Magendie, U1215, 33000 Bordeaux, France; 6 Department of Basic Sciences, Faculty of Medicine and Sciences, Universidad San Sebastián, Santiago de Chile, Chile; 7 Laboratorio de Hormonas y Regulación Metabólicas, Instituto de Nutrición y Tecnología de los Alimentos (INTA), Universidad de Chile, Santiago, Chile

**Keywords:** Endocannabinoid receptor, Skeletal muscle, Autophagy, High-fat diet

## Abstract

The endocannabinoid system (ECS) regulates energy metabolism, has been implicated in the pathogenesis of metabolic diseases and exerts its actions mainly through the type 1 cannabinoid receptor (CB1). Likewise, autophagy is involved in several cellular processes. It is required for the normal development of muscle mass and metabolism, and its deregulation is associated with diseases. It is known that the CB1 regulates signaling pathways that control autophagy, however, it is currently unknown whether the ECS could regulate autophagy in the skeletal muscle of obese mice. This study aimed to investigate the role of the CB1 in regulating autophagy in skeletal muscle. We found concomitant deregulation in the ECS and autophagy markers in high-fat diet-induced obesity. In obese CB1-KO mice, the autophagy-associated protein LC3 II does not accumulate when mTOR and AMPK phosphorylation levels do not change. Acute inhibition of the CB1 with JD-5037 decreased LC3 II protein accumulation and autophagic flux. Our results suggest that the CB1 regulates autophagy in the tibialis anterior skeletal muscle in both lean and obese mice.

**Supplementary Information:**

The online version contains supplementary material available at 10.1186/s40659-023-00426-5.

## Background

Obesity has become a pandemic in modern societies, and its treatment is a complex public health concern, being the fifth leading cause of death worldwide [1]. Approximately ~ 28 million people around the world die as a result of overweight or obesity co-morbidities, including hypertension, dyslipidemia, insulin resistance, stroke, diabetes mellitus, fatty liver disease, coronary heart diseases, cancer, and metabolic diseases [2, 3]. Lifestyle modifications, nutritional, surgical, and pharmacological therapeutic strategies have been used to fight this condition [4]. Macroautophagy (herein referred to as autophagy) and the endocannabinoid system (ECS) are involved in the progress of obesity. Autophagy is a lysosome-dependent catabolic process whereby misfolded proteins and organelles are degraded and recycled for multiple processes [5]. The aberrant behavior of basal autophagy contributes to the pathogenesis of several diseases, including cancer, cardiovascular diseases, obesity, diabetes mellitus, and aging. In particular, disrupted autophagy in skeletal muscle is associated with lipid droplet accumulation, muscle mass imbalance, and metabolic homeostatic alterations [6–9]. For instance, intact autophagy is essential for preserving muscle structure and fitness under basal conditions [10]. Masiero et al. showed that autophagy-incompetent muscle progressively degenerates due to aberrant proteostasis [7]. Conversely, the stimulation of autophagy induces beneficial effects, such as retarding the age-dependent loss of muscle mass [11].

The ECS encompasses a large group of endogenous molecules, and the most studied endocannabinoids include arachidonoylethanolamide (a.k.a. anandamide, AEA) and 2-arachidonoylglycerol (2-AG). Several enzymes are involved in their synthesis and degradation and two well-known G-protein coupled receptors, i.e., type 1 and type 2 cannabinoid receptors (CB1 and CB2) are involved in their signaling [12]. The main enzymes involved in the anandamide and 2-AG syntheses are the N-acyl phosphatidylethanolamine phospholipase D (Napepld) and diacylglycerol lipase alpha (Daglα), respectively. In addition, the identified hydrolyzing enzymes are fatty acid amide hydrolase (FAAH), which degrades anandamide, and monoacylglycerol lipase (MAGL), able to hydrolyze 2-AG. The CB1 is widely expressed in central and peripheral tissues, including the central nervous system, liver, adipose tissue, and skeletal muscle [13].

Diet-induced obesity leads to changes in the expression of the CB1, FAAH, and MAGL in several tissues and increased circulating endocannabinoids [14–16]. These changes affect the architecture and physiology of skeletal muscle, as previously described [17, 18]. From a general perspective, the ECS affects food intake and energy metabolism; for instance, pharmacological inhibition of CB1 by SR141716 (Rimonabant) reversed the obesity complications in rodents and improved several metabolic processes [19, 20]. Moreover, the CB1 is required for the development of diet-induced steatosis, dyslipidemia, and insulin and leptin resistance [21]. Treatment with a CB1 agonist increases de *novo* fatty acid synthesis in the liver or isolated hepatocytes, thus contributing to diet-induced obesity [15] and strongly suggesting a role in the progress of obesity and its comorbidities.

On the other hand, CB1 is known to influence mTOR and AMPK signaling (both known signaling pathways the regulate autophagy) [22, 23]. Although it is known that the CB1 participates in autophagy regulation in neurons [24, 25], it is unclear if the CB1 can modulate autophagy by canonical pathways in vivo in the tibialis anterior muscle. Furthermore, many studies have investigated the role of the ECS in regulating energy balance and metabolism in the nervous system and peripheral organs [14, 16, 26], however, very few have examined the physiological role in skeletal muscle and, specially, the control of relevant biological processes, such as autophagy. This study aims to investigate the role of the CB1 in regulating autophagy in the tibialis anterior muscle. Here, we show evidence that the CB1 regulates basal autophagy in the tibialis anterior muscle in normal and diet-induced obese mice.

## Results

### A high-fat diet affects the ECS and autophagy in the tibialis anterior muscle

**Fig. 1:**
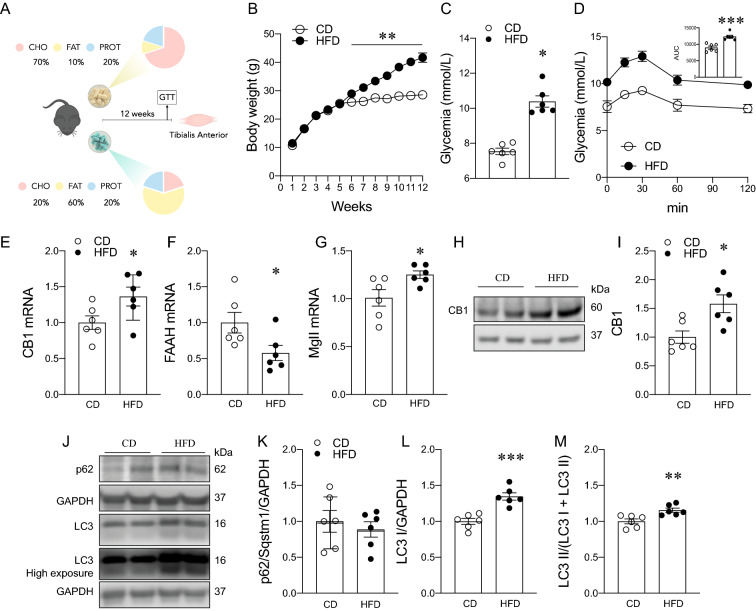
Effects of a high-fat diet on body weight, glucose homeostasis, endocannabinoid system, and autophagy markers. **A** Study design. **B** Body weight. **C** Glycemia. **D** Glucose tolerance test (GTT). **E** Expression of CB1 mRNA levels. **F** Expression of FAAH mRNA levels (fatty acid amide hydroxylase). **G** Expression of monoacylglycerol lipase (Mgll) mRNA levels. **H**–**I** CB1 protein levels. **J** Representative western blot. (K) p62/Sqstm1 protein levels. **L** LC3 I protein levels. **M** LC3 II normalized by total LC3. Two-way analysis of variance (ANOVA) repeated measurements with Bonferroni’s post hoc test and unpaired t-test were conducted. *p < 0.05; **p < 0.01; ***p < 0.0005; ****p < 0.0001. Values are expressed as mean and S.E.M. and scatter dot plots, as appropriate

As previously suggested, exposure to a high-fat diet (HFD) leads to the deregulation of several biological systems [27–29]. We focused on the tibialis anterior (TA) skeletal muscle due to previous reports showing that autophagy modulation in this muscle is key to maintaining muscle mass [7, 30, 31]. As we expected, body weight, fasting glycemia, and area under the curve (AUC) of an intraperitoneal glucose tolerance test were increased in mice fed with a HFD for 12 weeks (Fig. 1A–E; *p < 0.05; **p < 0.01; ***p < 0.001; and ****p < 0.0001). We found an increase in CB1 mRNA and protein levels in the TA in the HFD group (Fig. 1E–I; *p < 0.05). Moreover, mRNA expression was reduced for FAAH and increased for MAGL (Mgll), respectively, in the TA of mice fed with a HFD (Fig. 1F, G; *p < 0.05). To evaluate the effect of diet-induced obesity on autophagy markers, we then measured the protein levels of LC3 and p62/Sqstm1 (markers) in TA muscle. No significant changes were observed in the p62/Sqstm1 ratio. However, LC3 I and LC3 II accumulation was observed in the HFD group (Fig. 1J–M; **p < 0.01 and ***p < 0.001). These results suggest that diet-induced obesity increases ECS activity and LC3 II protein levels.

### Deletion of the CB1 prevents LC3 II accumulation induced by a high-﻿fat diet in the tibialis anterior muscle

**Fig. 2:**
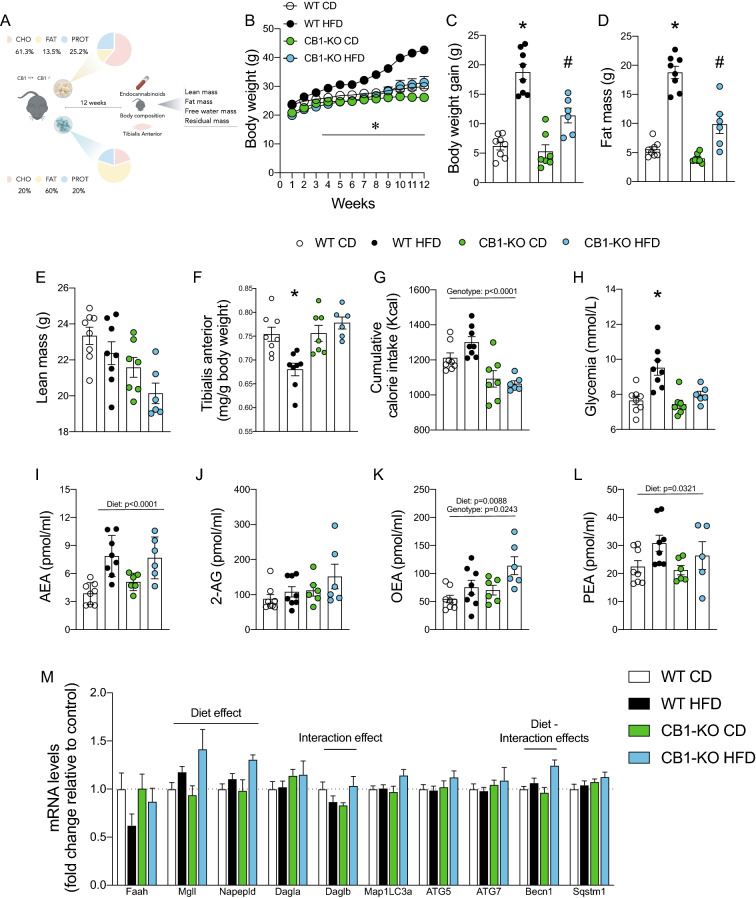
Knockout of CB1 prevents impairment induced by a high-fat diet. **A** Study design. **B** Body weight curve. **C** Body weight gain. **D** Fat mass. **E** Lean mass. **F** Tibialis anterior weight. **G** Cumulative calorie intake. **H** Glycemia. **I** Plasma anandamide (AEA). **J** 2-arachidonoylglycerol (2-AG). **K** N-palmitoyl-ethanolamine (PEA). **L** Oleoylethanolamine (OEA). **M** mRNA levels. Two-way analysis of variance (ANOVA) repeated measurements with Bonferroni’s post hoc test. Statistical significance was set at p < 0.05. *WT HFD vs. all groups. Two-way analysis of variance (ANOVA) with Bonferroni’s post hoc test. * WT HFD vs. all groups. # CB1-KO HFD vs WT CD and CB1-KO CD. Statistical significance was set at p < 0.05. Values are expressed as mean and S.E.M. and scatter dot plot, as appropriate

**Fig. 3:**
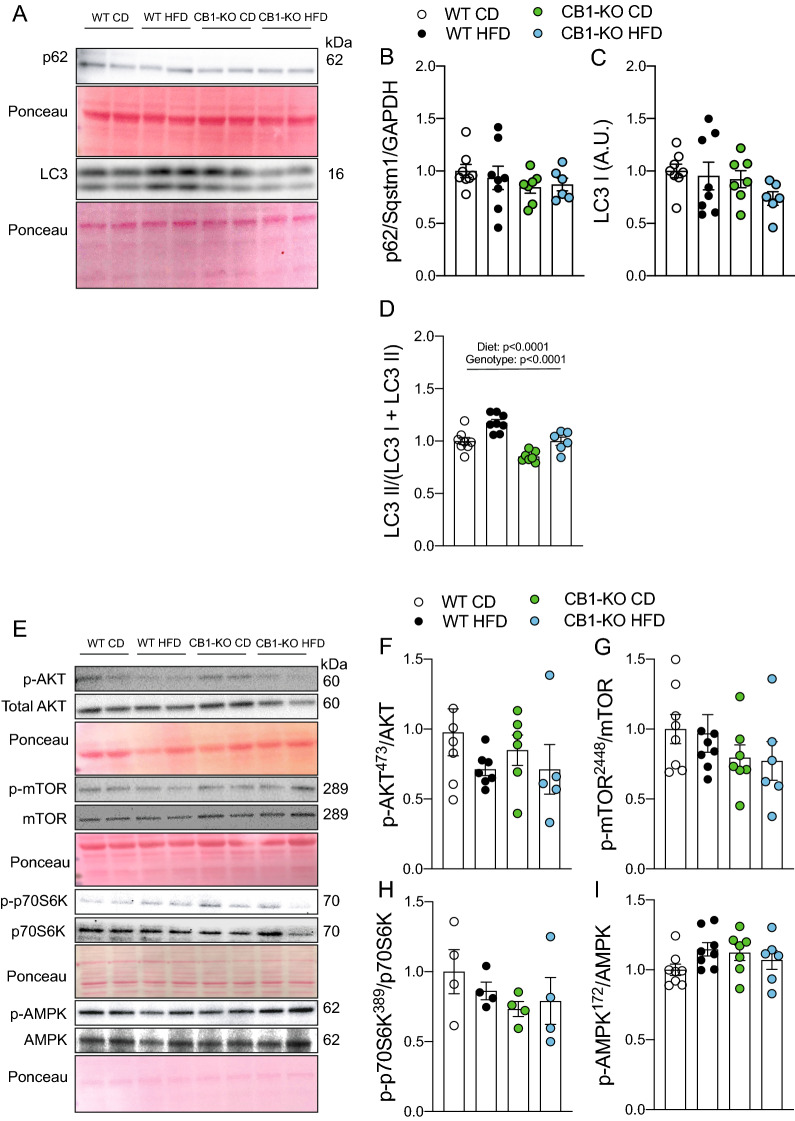
Knockout CB1 prevents LC3 II accumulation in tibialis anterior muscle. p62/Sqstm1, LC3, AKT^473^, total AKT, mTOR^2448^, total mTOR, p70S6K^389^, total p70S6K, AMPK^172^, and total AMPK protein levels were determined by western blot. **A** Representative western blot image. **B** p62/Sqstm1 protein levels. **C** LC3 I protein levels. **D** LC3 II normalized by total LC3. **E** Representative western blot image. **F** AKT^473^. **G** mTOR^2448^. **H** p70S6K^389^. **I** AMPK^172^. Two-way analysis of variance (ANOVA) with Bonferroni’s post hoc test. Statistical significance was set at p < 0.05. Values are expressed as mean and S.E.M. and scatter dot plots, as appropriate

Since we observed concomitant deregulation in the ECS and basal autophagy markers, we wanted to determine if the deletion of the CB1 prevented LC3 II accumulation induced by a HFD in the TA. We fed CB1-KO male mice with a control or a HFD for 12 weeks (Fig. 2A). Genetic deletion of CB1 was confirmed by western blot analysis of TA (Supp. 1A). As previously reported [32], we found that CB1-KO mice were protected from diet-induced obesity, compared to WT controls, in terms of body weight, fat mass, and tibialis anterior weight,with no changes in lean mass (Fig. 2B–F). There was a lower cumulative calorie intake effect in mice with deletion of CB1 (Fig. 2G). HFD increased plasma glucose levels, but CB1 deletion prevented this hyperglycemia (Fig. 2H). Next, we evaluated endocannabinoids plasma concentrations. Mice fed with a HFD increased AEA and PEA plasma levels (Diet: p < 0.0001). Moreover, OEA levels increased by genotype and diet effect (Diet: p = 0.0088; Genotype: p = 0.0243) (Fig. 2I–L). We then explored the effect of diet-induced obesity on the mRNA expression of genes of the endocannabinoid system and autophagy in the TA. As shown in Fig. 3M, Mgll and Napepld revealed a diet effect (p < 0.05), suggesting an increase of both enzymes. An interaction effect (p < 0.05) was found in Daglβ, showing restoration of mRNA levels in HFD-fed CB1-KO mice. Finally, Becn1 had a diet and interaction effect (p < 0.05), indicating that a HFD increases its mRNA expression levels, and that the genotype further increases it.

To investigate whether CB1 knockout prevented LC3 II accumulation induced by a high-fat diet, we determined autophagy-related proteins levels. No significant changes were observed in p62/Sqstm1 and LC3 I protein levels (Fig. 3A–C). Interestingly, a diet and genotype effect (p < 0.0001) was found in LC3 II (Fig. 3D), suggesting a role for the CB, independently of the diet. To further elucidate if the genotype effect of LC3 II accumulation was driven by changes in the regulator proteins of autophagy, we evaluated the levels of AMPK and AKT/mTOR/p70S6K phosphorylation, which, unexpectedly, did not change (Fig. 3E–I).

### The CB1 regulates basal autophagy

**Fig. 4:**
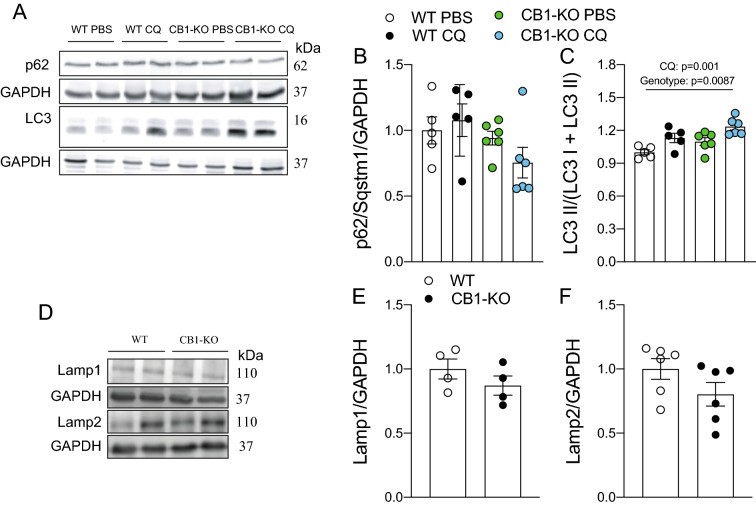
The CB1 regulates basal autophagy in tibialis anterior muscle. p62/Sqstm1, LC3, Lamp1, and Lamp2 protein levels were determined by western blot. **A** Representative western blot image. **B** p62/Sqstm1 protein levels. **C** LC3 II is normalized by total LC3. **D** Representative western blot image. **E** Lamp1 protein levels. **F** Lamp2 protein levels. Two-way analysis of variance (ANOVA) with Bonferroni’s post hoc test and unpaired *t*-test were conducted. Statistical significance was set at p < 0.05. Values are expressed as mean and S.E.M. and scatter dot plots, as appropriate

**Fig. 5:**
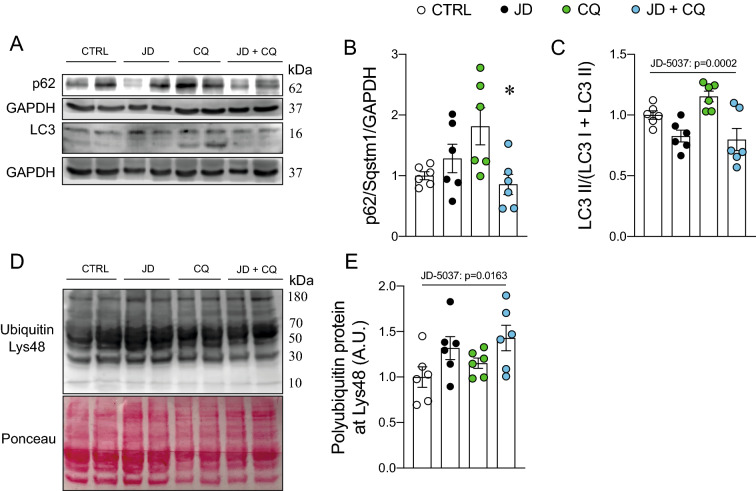
JD-5037 reduces LC3 II protein in vivo. p62/Sqstm1, LC3, and polyubiquitin protein levels were determined by western blot. **A** Representative western blot image. **B** p62/Sqstm1 protein levels. **C** LC3 II is normalized by total LC3. **D** Representative western blot image. **E** Polyubiquitin protein levels. A two-way analysis of variance (ANOVA) with Bonferroni’s post hoc test was conducted. Statistical significance was et al. p < 0.05. *CQ vs. JD + CQ. Values are expressed as mean and S.E.M. and scatter dot plot, as appropriate

Due to the surprising absence of changes in the protein phosphorylation of AMPK and the mTOR pathway, we evaluated whether the CB1 regulated autophagic flux by using chloroquine in WT and CB1-KO mice. We found no difference in p62/Sqstm1 protein levels (Fig. 4A, B). As we expected, chloroquine induced LC3 II accumulation (CQ: p = 0.001 and Genotype: p = 0.0087, Fig. 4C). To explore whether changes in the LC3 proteins in the second experiment with the HFD were the results of alterations in the lysosome, we then evaluated the Lysosomal-associated proteins 1 (Lamp1) and 2 (Lamp2), but there were no changes (Fig. 4D–F). To determine if pharmacological inhibition of the CB1 increased autophagic flux, we injected C57 mice intraperitoneally with a dose of JD-5037 (3 mg kg^−1^ body weight), which is a CB1 antagonist with low brain penetration [33]. An interaction effect was found in the JD + CQ group in p62/Sqstm1 protein levels (Interaction: p = 0.0085; Fig. 5A, B). Regarding LC3 proteins, JD-5037 produced an effect (p = 0.0002), suggesting that pharmacological blockade of CB1 prevents chloroquine-induced changes in autophagic flux. Lastly, we observed an accumulation of polyubiquitinated protein in mice injected with JD-5037 (JD-5037 effect: p = 0.0163). These results suggest that while genetic deletion of CB1 does not regulate autophagic flux, acute pharmacological inhibition of the CB1 with JD-5037 decreases LC3 II protein accumulation and autophagic flux.

## Discussion

In this study, we investigated the role of CB1 in regulating autophagy in the TA skeletal muscle. We found an association between ECS deregulation and impaired basal autophagy in mice fed with a HFD. The alterations of the ECS were mainly characterized by a rise in the CB1 and a reduction of FAAH. On the other hand, interestingly, these results may constitute a concerted mechanism to activate CB1 with anandamide, its natural agonist. In addition, LC3 II accumulation is the distinctive point of autophagy impairment. We observed that the deletion of the CB1 in mice fed with a HFD prevented LC3 II accumulation, supporting the role of CB1 in favoring the increase of LC3 II observed in HFD. However, there was no change in the phosphorylation levels of the AMPK and mTOR pathways, two critical regulatory pathways of autophagy. Finally, while genetic deletion of CB1 regulated autophagy, acute pharmacological inhibition of the CB1 with JD-5037 decreased LC3 II protein accumulation and autophagic flux.

Previous studies have found an increase in CB1 levels in the liver, adipose tissue, and central nervous system of mice and humans with obesity [15, 34–37]. Accordingly, we found that CB1 mRNA and protein levels were increased in the TA of mice fed with a HFD. This fact could be relevant to muscle metabolism and physiology. Esposito et al. [18] exposed differentiated L6 skeletal muscle cells to SR141716 (Rimonabant), a known CB1 antagonist/inverse agonist, increasing 2-deoxyglucose uptake in a time- and dose-dependent manner. A similar effect was also induced by gene silencing of the CB1. In accordance, Liu et al. [38] treated female mice with a daily i.p. dose of Rimonabant, increasing glucose uptake by 68% in an isolated soleus muscle preparation. In addition, oxygen consumption was higher in Rimonabant- compared with vehicle-treated muscle. These reports corroborate the significant role of CB1 in muscle metabolism and physiology. Additionally, we found decrease FAAH mRNA expression, suggesting reduced activity and, as a result, a possible rise of anandamide at the plasmatic and tissue levels, which may lead to persistent receptor activation [39]. Sipe et al. [40] studied a missense polymorphism in FAAH in 2667 subjects of white, black, and Asian ancestry. Their results indicate that FAAH polymorphism is a risk factor in the overweight/obese population. A role for this enzyme in energy homeostasis has also been shown in FAAH-deficient (FAAH^(−/−)^) mice. These FAAH^(−/−)^ animals had increased total adipose tissue and body weight, and increased levels of anandamide in several tissues [41].

Aberrant autophagy has been reported in several tissues in obese mice [6, 42, 43]. Our findings show that basal autophagy is affected by diet-induced obesity in the tibialis anterior. Furthermore, decreased muscle mass has been found in mice fed with a HFD [44], and CB1-KO mice were protected from this effect. LC3 II accumulation correlates with either enhanced autophagosome synthesis or reduced autophagosome turnover, probably due to delayed trafficking to the lysosome, reduced fusion between compartments, or impaired lysosomal proteolytic activity [45]. Fan and Xiao [46] showed that autophagy was compromised in the muscle of rats fed with a HFD. The authors fed rats with an HFD and then treated them with the autophagy inhibitor chloroquine. Using immunostaining and western blot, they found that the LC3 II/I ratio was increased in the muscle of rats fed with a HFD, denoting impaired autophagy. In agreement, Li et al. [47] showed that a HFD inhibits the production of autophagic lysosomal expression of autophagy-related genes (LC3 II, Becn1, and ATG5) and increases the accumulation of p62/Sqstm1 in the gastrocnemius muscle. Our findings show that basal autophagy is affected by diet-induced obesity. Therefore, our results suggest concomitant impairments in the ECS and autophagy in the TA.

Autophagy is a conserved cellular degradation process in which parts of the cytosol and organelles are sequestered into an autophagosome and delivered into a lysosome for breakdown and eventual recycling of the resulting macromolecules. Genetic deletion of the CB1 prevented the LC3 II accumulation induced by a HFD. We can speculate that decreased LC3 II levels reflect the restoration of autophagy, an explanation that requires further study. Unexpectedly, mTOR and AMPK, the two multiprotein complexes involved in the canonical induction pathway of autophagic vesicle formation, do not seem to be altered in our experimental setting. Autophagy is also regulated by non-canonical pathways that lead to autophagosomal degradation [48]. These alternative pathways are stimuli and cell-type dependent, thus future studies are required to better understand the mechanism that leads to this non-canonical autophagy regulation.

Autophagic flux assay is considered the best characterization of autophagy levels [49, 50]. Hence, we performed an autophagic flux assay in vivo in CB1-KO mice treated with an i.p. injection of chloroquine. We found higher LC3 II accumulation in wild-type and CB1-KO mice previously treated with chloroquine and, unexpectedly, lack of the CB1 did not affect LC3 II abundance. However, mice treated only with a CB1 antagonist, JD-5037, reduced LC3 II basal levels. Furthermore, JD-5037 reverted LC3 II accumulation by blocking chloroquine-induced fusion of the autophagosome with lysosomes. As mentioned above, our data suggest that CB1-KO prevents LC3 II accumulation, indicating restoration of the autophagy in mice fed a HFD. However, CB1-KO does not impact autophagic flux, suggesting that there is a compensation mechanism in KO mice; moreover, this experiment was not done in mice fed with a high-fat diet with ECS overactivation. An alternative explanation is that the CB1 regulates LC3 lipidation through competitive anandamide production. NAPE-PLD requires calcium for the anandamide synthesis route, with phosphatidylethanolamine (PE) as a source [51]. PE is also a substrate for LC3 II production. It is known that CB1 regulates intracellular calcium flux [52]. Oláh et al. showed that the activation of the CB1 inhibits sarcoplasmic Ca^2+^ release and the sarcoplasmic reticulum Ca^2+^ ATPase during excitation–contraction coupling in a G_i/o_ protein-mediated manner in adult skeletal muscle fibers. This could, in part, explain the reduction of LC3 II protein levels in mice injected with JD-5037; however, more research is necessary to confirm this hypothesis. Due to a possible effect on lysosomal function, we evaluated two lysosomal proteins, Lamp1 and Lamp2. Similarly, there was no difference between wild-type and CB1 KO, suggesting that the lysosome was unaffected. We also evaluated polyubiquitinated proteins at specific lysine residues (Lys48) because it labels proteins for proteasomal degradation. Interestingly, more polyubiquitinated proteins were found in mice treated with JD-5037. This result is consistent with the reduction of LC3 II protein, but the mechanism behind this is still elusive.

Contrary to our findings, Hiebel et al. [53] showed that siRNA knockdown of CB1 activity affects the autophagic flux independent of the canonical pathway. They performed an autophagic flux assay in human embryonic kidney cells, showing LC3 II accumulation, suggesting a possible role of the CB1 in autophagy regulation. Piyanova et al. [54] treated with Rapamycin (a known mTOR inhibitor that induces autophagy) and Baf1 (to inhibit autophagic flux) to hippocampal neurons from CB1-KO. LC3 II levels were higher in Cnr1^−/−^ neurons compared with Cnr1^+/+^, indicating that the autophagic rate is higher in the absence of CB1. Moreover, Blázquez et al. [55] treated wild-type mice with a single i.p. injection of the Δ^9^-tetrahydrocannabinol (THC), which exerts its biological effects mainly by activating the CB1, and evaluated LC3 protein. LC3 II levels were increased, suggesting that THC impairs the autophagy and that this process occurs selectively in the striatum. Genetic background, mouse strain, diet composition, mice age, and cell line types could explain these discrepancies with our results. Additionally, TA in C57BL/6 mice is a fast-contracting muscle with a high percentage of type IIB fibers [56]. We explored mRNA levels of autophagy proteins but did not find alterations that could explain the reduction of LC3 protein levels. Additional autophagy or lysosomal genes and proteins should be assessed to elucidate these results.

Restoring autophagy levels in several diseases is key to recovering physiological processes. Autophagy is a potential target for developing pharmacological and non-pharmacological interventions to manage obesity. Therefore, it is relevant to search for new pharmacological targets to counteract obesity-related disruptions in different physiological systems. Pharmacological blockers of the CB1, such as Rimonabant, were promising agents to reduce food intake and body weight and revert the metabolic alterations induced by obesity. However, its negative side effects related to severe complications of neuropsychiatric effects (i.e. anxiety, depression, and suicidal ideation) [57] led to its withdrawal from the market. The design of new CB1 peripheral blockers could remove these unwanted effects. JD-5037 exerts a positive impact on the control of body weight, metabolic outcome, and low brain penetration without adverse side effects [33]. However, in the current model, we observed that the inhibition of CB1 with JD-5037 produces a reduction of autophagic flux that needs further investigation.

It has been widely reported that obesity induces sarcopenia. In our results, CB1-KO prevents weight reduction in TA induced by a high-fat diet. Iannotti et al. (2014) showed in human and mouse myoblasts that the stimulation of CB1 with 2-AG or arachidonyl‐2′‐chloroethylamide prevents myotube formation and promotes myoblast proliferation. Interestingly, these effects are reverted with rimonabant [17]. Moreover, Iannotti et al. (20,018) showed that treatment with rimonabant promotes human satellite cell differentiation in vitro and increases the number of myofibers [58]. However, Le Bacquer et al. (2022) co-incubated C2C12 myotubes with dexamethasone and rimonabant for 24 h, and the atrophy was prevented in myotubes exposed to rimonabant, without affecting the atrogin-1/MAFbx ratio. The authors also found that rimonabant stimulates protein synthesis and CB1 agonists are unable to modulate protein synthesis, suggesting a CB1-independent mechanism [59]. In vitro and in vivo models, mouse strains, and rimonabant doses can explain these controversial results.

## Conclusions

The present investigation provides evidence of altered autophagy in diet-induced obesity and increased “endocannabinoid tone.” Our results also indicate a regulatory role of the CB1 on fast muscle, such as the tibialis anterior in mice, reducing LC3 II accumulation induced by a HFD. In addition, acute pharmacological inhibition of the CB1 reduces LC3 II accumulation induced by chloroquine, suggesting a reduction of autophagic flux. In conclusion, CB1 regulates autophagy in the tibialis anterior muscle in mice. Although our study could not clarify why and how CB1 regulates autophagy, it opened a new area of research.

## Methods

### Animals and diet

All animal study protocols were reviewed and approved by the local Committee on Animal Health and Care of the University of Bordeaux and the Institute of Nutrition and Food Technology. For the first experiment, male C57BL/6 littermate mice were kept with food and tap water ad libitum*,* and a 12–12 h light–dark cycle. Mice were fed with a control diet (CD, n = 6) containing 70% carbohydrates, 10% fat, and 20% protein in terms of kcals (Research Diet Inc. D12450J, New Brunswick, NJ, USA) and a high-fat diet (HFD, n = 6) containing 60% fat (90% lard, 10% soybean oil), 20% carbohydrates and 20% protein in terms of kcal (D12492, Research Diets, New Brunswick, NJ, USA). Body weight and food intake were assessed weekly. At the end of the experiment, a glucose tolerance test (GTT) was performed. Finally, mice were euthanized, and the tibialis anterior (TA) muscle was removed for analysis.

For the second experiment, male C57BL/6 CB1^+/+^ (WT) and CB1^–/–^ (CB1-KO) littermate mice were maintained under standard conditions (food and tap water ad libitum; 12 h–12 h light–dark cycle). Mice were randomly assigned into 4 groups: WT CD (Wild-type control diet, n = 8), WT HFD (Wild-type high-fat diet, n = 8), CB1-KO CD (CB1 knockout control diet, n = 7), and CB1-KO HFD (CB1 knockout high-fat diet, n = 6). The mice were fed for 12 weeks with a chow diet containing 13.5% fat, 61.3% carbohydrates, and 25.2% protein in kcal (SAFE, A03 SP/17275) or a high-fat diet (D12492, Research Diets, New Brunswick, NJ, USA). Body weight and food intake were measured each week up to the end of the experiment. Body composition was also evaluated during the first and 12 week by nuclear echo magnetic resonance imaging whole-body composition analysis (EchoMRI 900; EchoMedical Systems, USA), as described previously [60]. Glycemia was measured in mice fasting for 6 h. Finally, the mice were sacrificed the next day after measuring the glycemia, and the TA was collected and frozen (− 80 °C). For the third experiment, mice were grouped in: WT PBS (Wild-type Phophate-Buffered Saline; n = 5), WT CQ (Wild-type chloroquine; n = 5), CB1-KO PBS (n = 6), and CB1-KO CQ (n = 7). They fasted for 24 h and were subjected to an intraperitoneal injection of CQ (100 mg kg body weight) or PBS 4 h before sacrifice. Animals were sacrificed, and the TA muscle was quickly dissected and frozen for immunoblot analyses. For the fourth experiment, male C57BL/6 littermate mice were grouped in: CTRL (Control; n = 6), CQ (chloroquine; n = 6), JD (JD-5037; n = 6), and JD-CQ (n = 6). Mice were kept in fasting condition for 24 h. 4 h before sacrifice, mice were injected with 100 mg kg body weight of chloroquine or PBS. After 2 h, an intraperitoneal injection of 3 mg kg^−1^ body weight of JD-5037 or vehicle (1% DMSO, 1% Tween80, and PBS was applied. Finally, the animals were sacrificed, and the TA muscle was quickly dissected and frozen for immunoblot analyses.

### Glucose tolerance test

Three days before sacrifice, a glucose tolerance test was performed in the morning. Mice fasted for 6 h in separated individual cages. Basal blood glucose was measured in samples collected from the tail with an Accu-Check Performa glucometer (Roche Diagnostic, Mannheim, Germany). Then, 1.5 mg kg^−1^ body weight of glucose in PBS was injected intraperitoneally, and blood glucose was evaluated at 15, 30, 90, and 120 min time points. At the end of the assessment, mice were placed in their cages.

### Endocannabinoid quantification

To determine endocannabinoids, we used the liquid chromatography-tandem mass spectrometric analysis method [61]. The blood samples of mice were collected and homogenized with chloroform/methanol (2:1, v/v) containing internal deuterated standards (Cayman Chemicals, Ann Arbor, MI, USA). Purified endocannabinoids were then evaluated by isotopic dilution, according to a calibration curve.

### RNA extraction and PCR procedure

Total RNA was extracted from samples using a commercial kit (Fermentas, Villebon sur Yvette, France). Random hexamer primers and Oligo(dt)18 primers (Fermentas) were used for cDNA synthesis from 2 μg of total RNA with RevertAid Premium Reverse Transcriptase (Fermentas). Real-time PCR was performed using transcript-specific primers, cDNA (1 ng), and LightCycler 480 SYBR Green I Master (Roche) in a final volume of 10 μl. The 2 − ΔΔCT method was used for relative quantification analysis, and PCR amplification of the housekeeping genes Ywhaz, Gapdh, and Tubulin alpha was used for controls. The primer’s sequences are reported in Additional file 1: Table S1.

### Western blot analyses

Total protein lysates were prepared using T-PER lysis buffer (78,510, ThermoScientific) with phosphatases/protease inhibitors. Lysates were centrifuged at 12,000 rpm for 10 min at 4 °C. Total protein concentrations were measured using the BCA assay kit (71,285–3, Novagen^®^, Merck, MA USA). Proteins were separated using 6–15% SDS-PAGE and transferred to PVDF membranes. The membranes were blocked with 5% low-fat milk (Svelty, Nestlé) in T-TBS/0.1% (1 mM Tris) Tween-20 and incubated overnight at 4 °C with primary antibodies. The membranes were then extensively washed with TBS/0.1% Tween-20, and incubated with the secondary polyclonal anti-mouse (#402335, Calbiochem) or anti-rabbit (#401315, Calbiochem) antibodies. The protein bands in the blots were visualized using HRP secondary antibodies. Enhanced chemiluminescence ECL-plus reagent (DW1029, Biological Industries) and C-DiGit^®^ Blot Scanner (LI-COR, USA) were used and bands were analyzed with the Image J version 1.51 software (National Institutes of Health, Bethesda, MD, USA). The primary antibodies used were CB1 (Cayman #10006590), LC3A/B (CST #4108), p62/Sqstm1 (NBP1-48320, Novus), GAPDH (CST #5174), phospho-p70S6K Thr389 (CST #9234), p70S6K Total (CST #2708), phospho-AMPKα Thr172 (CST #2535), AMPKα Total (CST #5831), phospho-Akt Ser473 (CST #9271), Akt Total (CST #9272), phospho-mTOR Ser2448 (CST #2971), mTOR total (CST #2972), Lamp1 (CST #3243), Lamp2 (CST #49,067), and K48-linkage Specific Polyubiquitin (CST # 4289). GAPDH or total protein content were used as loading controls.

### Statistical analysis

All values are presented as mean ± standard error of the mean (SEM). Statistical analyses were performed using Student's t-test, repeated measure analysis of variance (ANOVA), and two-way ANOVA for non-repeated measures. Diet—genotype, genotype—chloroquine, and JD-5037—chloroquine were used as the main effects for the variables. Bonferroni’s post hoc test was applied where appropriate, and statistical significances were set at p < 0.05. All statistical analyses were performed using the GraphPad Prism software (version 8.0, San Diego, CA, USA).

## Supplementary Information

**Additional file 1: **Representative western blot image.

## Data Availability

The data that support the findings of this study are available from the corresponding authors (C.S. and R.T.) upon reasonable request.

## Abbreviations

2-AG: 2-Arachidonoylglycerol
AEA: Arachidonoylethanolamide or anandamide
CB1: Type 1 cannabinoid receptor
CD: Control diet
CQ: Chloroquine
Daglα: Diacylglycerol lipase alpha
Daglβ: Diacylglycerol lipase beta
ECS: Endocannabinoid system
FAAH: Fatty acid amide hydrolase
GTT: Glucose tolerance test
HFD: High-fat diet
KO: Knockout
Lamp1: Lysosomal-associated protein 1
Lamp2: Lysosomal-associated protein 2
LC3-I: Microtubule-associated protein 1 light chain 3
LC3-II: Phosphatidylethanolamine conjugated form of microtubule associated protein 1 light chain 3
MAGL: Monoacylglycerol lipase
Napepld: N-acyl phosphatidylethanolamine phospholipase D
OEA: Oleoylethanolamine
PEA: N-palmitoyl-ethanolamine
PE: Phosphatidylethanolamine
TA: Tibialis anterior
WT: Wild type
