# Supplementary material for: The CB1 cannabinoid receptor regulates autophagy in the tibialis anterior skeletal muscle in mice
Source: Biol Res. 2023 Mar 25;56:14. doi: 10.1186/s40659-023-00426-5 (PMC10039507; doi:10.1186/s40659-023-00426-5)
Supplement: Supplementary file 1 — Additional file 1: Representative western blot image. [file 40659_2023_426_MOESM1_ESM.pdf]

Additional file 1: Fig. S1

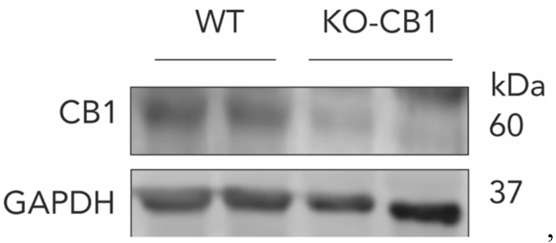

,

Additional file 1: Table. S1: Quantitative real time PCR primers

Gene	GenBank ID	Sense	Antisense
Cnr1	NM_007726	GTGCTGTTGCTGTTCATTGTG	CTTGCCATCTTCTGAGGTGTG
Faah	NM_010173	CGGCCATCTTGAGGGTCAT	GGCTGCAGTGCAGAGCG
Mgll	NM_011844	TCACACTTCCCTTTCTCTCCTGAT	GTGACAAACCAGTGACCCACTGT
Napepld	NM_178728	GAACGGCCTTGGCATAGCT	GACCAGACCACAAATCACATGG
Dagla	NM_198114	GGTCCTGCTCGTGCTGTCTC	TGCAGCCACAACAGTTGTCTTC
Daglb	NM_144915	TTGTAGGCCAGCCCATGG	GCCTTGCAGACCCACTAAGG
Map1lc3a	NM_025735	ACACCCATCGCTGACATCTATG	TGGGAGGCGTAGACCATGTAG
Atg5	NM_053069	CCATTTCTCACTGGCATAGCAA	TAATAAATCCTGTGACAGCTCCTGA
Atg7	NM_001253717	GGACACCCCAGGACCCA	CCAGAGCAGGTGCCCTACC
Becn1	NM_019584	GTCTTCCGTACAGGATGGACGT	GGAATAGGAGCCGCCACTG
Ctsb	NM_007798	GAAGAAGCTGTGTGGCACTG	GTTCGGTCAGAAATGGCTTC
Tfeb	NM_011549.3	GACTCAGAAGCGAGAGCTAACA	TGTGATTGTCTTTCTTCTGCCG
Sqstm1	NM_011018	TGCTGACGCCTGAGAACAGA	GCCAGGCCTAGGGAAAGC
Gapdh	NM_001289745.3	TGAAGGTCGGAGTCAACGGATTTG GT	CATGTGGGCCATGAGGTCCACCAC
Ywhaz	NM_001135699	AGCCATTGCTGAACTTGATACA	AATTTTCCCCTCCTTCTCCTG
Tuba4a	NM_009447	CCACTTCCCCTTGGCTACCTA	CCACTGACAGCTGCTCATGGT
